# Integration of Different “-omics” Technologies Identifies Inhibition of the IGF1R-Akt-mTOR Signaling Cascade Involved in the Cytotoxic Effect of Shikonin against Leukemia Cells

**DOI:** 10.1155/2013/818709

**Published:** 2013-06-19

**Authors:** Benjamin Wiench, Yet-Ran Chen, Malte Paulsen, Rebecca Hamm, Sven Schröder, Ning-Sun Yang, Thomas Efferth

**Affiliations:** ^1^Institute of Pharmacy and Biochemistry, Department of Pharmaceutical Biology, Johannes Gutenberg University, Staudinger Weg 5, 55128 Mainz, Germany; ^2^Agricultural Biotechnology Research Center, Academia Sinica, 115 Taipei, Taiwan; ^3^Cytometry Core Facility, Institute of Molecular Biology, Ackermannweg 4, 55128 Mainz, Germany; ^4^HanseMerkur Center for Traditional Chinese Medicine at the University Medical Center Eppendorf, 20246 Hamburg, Germany

## Abstract

Hematological malignancies frequently have a poor prognosis and often remain incurable. Drug resistance, severe side effects, and relapse are major problems of currently used drugs, and new candidate compounds are required for improvement of therapy success. The naphthoquinone shikonin derived from the Chinese medicinal herb, *Lithospermum erythrorhizon*, is a promising candidate for the next generation of chemotherapy. The basal cellular mechanism of shikonin is the direct targeting of mitochondria. Cytotoxicity screenings showed that the compound is particularly effective against leukemia cells suggesting an additional cellular mechanism. mRNA and miRNA microarrays were used to analyze changes in gene expression in leukemia cells after shikonin treatment and combined with stable-isotope dimethyl labeling for quantitative proteomics. The integration of bioinformatics and the three “-omics” assays showed that the PI3K-Akt-mTOR pathway was affected by shikonin. Deregulations of this pathway are frequently associated with cancerogenesis, especially in a wide range of hematological malignancies. The effect on the PI3K-Akt-mTOR axis was validated by demonstrating a decreased phosphorylation of Akt and a direct inhibition of the IGF1R kinase activity after shikonin treatment. Our results indicate that inhibiting the IGF1R-Akt-mTOR signaling cascade is a new cellular mechanism of shikonin strengthening its potential for the treatment of hematological malignancies.

## 1. Introduction

Hematological malignancies comprise different types of cancers that affect blood cells, bone marrow, or lymph nodes. According to the National Cancer Institute, leukemia, lymphoma, and myeloma accounted for 9% of all deaths from cancer in the United States in 2011. In recent years improvements in radio therapy and new therapeutics including imatinib [1], rituximab [2], and lenalidomide [3] clearly improved the response and survival rate of these diseases [4]. Despite this considerable advances in chemotherapy, drug resistance and relapse remain major problems and several hematological malignancies remain incurable with standard treatments [4, 5]. This lack of cures requires novel targeted and less toxic therapies, and new candidate compounds need to be tested for the improvement of therapy success. 

The naphthoquinone pigment shikonin is the most important pharmacologically active substance in the dried root of *Lithospermum erythrorhizon*. In traditional Chinese medicine (TCM) root extracts of *Lithospermum erythrorhizon* have been used to treat macular eruption, measles, sore-throat, carbuncles, and burns [6]. We recently showed that the natural compound shikonin has a strong cytotoxic effect against different cancer cell lines, especially sensitive and resistant leukemia cells. Shikonin directly targets mitochondria of cancer cells causing an overproduction of reactive oxygen species (ROS), mitochondrial dysfunction, and ultimately apoptosis [7]. However, the question why shikonin is particularly effective against leukemia and lymphoma cells remains unresolved.

In the present study, we investigated the effect of shikonin on the myeloid leukemia cell line U937 by the integration of different quantitative “-omics” technologies, combining high-throughput techniques as a promising tool for elucidating molecular mechanisms of new drugs in a fast and precise manner [8]. The integration of genomic and pharmacological analysis significantly accelerates the identification of cancer-specific synthetic lethal targets [9]. We analyzed the mRNA, miRNA, and protein expression in U937 cells after shikonin treatment and integrated the results using a bioinformatic approach. Thereby, it was possible to identify cellular functions and signaling pathways strongly deregulated after shikonin treatment. The data obtained from the proteomic and transcriptomic studies confirmed previous findings indicating that shikonin has strong effects on cell proliferation, cell cycle progression, cellular movement, and DNA integrity of cancer cells [7]. Interestingly, our findings indicated that one of the most affected signaling pathways in U937 leukemia cells was the phosphatidylinositol 3-kinase (PI3K)-Akt-mammalian target of rapamycin (mTOR) cascade. Hence, we proposed that an inhibition of this signaling network is a reason for the strong activity of shikonin against leukemia cells. We validated the effect of shikonin on the PI3K-Akt-mTOR pathway by demonstrating a decreased phosphorylation and activation of Akt after shikonin treatment using phospho-specific antibodies and flow cytometric analysis. In addition, kinase activity tests revealed that shikonin inhibits the kinase activity of the insulin-like growth factor 1 receptor (IGF1R), which is an important trigger of the PI3K-Akt-mTOR signaling cascade.

Targeting of PI3K-Akt-mTOR signaling became an attractive therapeutic strategy for cancer chemotherapy over the last few years [10, 11]. The signaling pathway plays a central role in cellular growth and survival through the regulation of protein synthesis and ribosomal protein translation [12]. Deregulations of mTOR signaling are associated with tumorgenesis, angiogenesis, tumor growth, and metastasis [10, 13]. The mTOR signaling pathway has been found to be frequently deregulated, especially in a wide range of hematological malignancies [14]. The signaling cascade is activated by receptor tyrosine kinases (RTKs, e.g., IGF1R and epidermal growth factor receptor (EGFR)), integrins, and cytokine receptors coupling external signals from growth factors, cytokines and the availability of nutrients to cell growth and proliferation [15]. After binding of the corresponding ligands, the RTKs activate PI3K, which in turn causes the phosphorylation of Akt. Activated Akt inhibits the heterodimeric complex of tuberous sclerosis proteins 1 and 2 (TSC1/2) that negatively regulates the mammalian target of rapamycin complex 1 (mTORC1) [16]. This complex is a centerpiece of the signaling cascade that controls protein synthesis by phosphorylation of different effector proteins, for example, the S6 kinase 1 (S6K1) and the 4E-binding protein 1 (4E-BP1) [17]. Much less is known about the second mTOR complex mTORC2. This complex responds to growth factors and regulates cell survival and metabolism, as well as the cytoskeleton [17]. Currently used drugs targeting this pathway are rapamycin and its derivatives (rapalogs) that directly target the mTORC1 complex [18, 19]. One weak point of these drugs is a resistance mechanism of cancer cells, which leads to an upregulation of IGF1R after mTORC1 inhibition [20–22]. This feedback mechanism causes an activation of the PIK3K-Akt-mTOR signaling cascade after initial inhibition resulting in only modest anticancer effects of rapalogs [14].

Ultimately, our results suggest that inhibition of IGF1R-Akt-mTOR signaling plays a key role in the cytotoxic effect of shikonin against U937 leukemia cells. Since this signaling network is frequently deregulated in hematological malignancies, shikonin is a promising candidate for the next generation of chemotherapy against these diseases.

## 2. Results

### 2.1. Cytotoxic Effect of Shikonin on U937 Leukemia Cells

**Figure 1:**
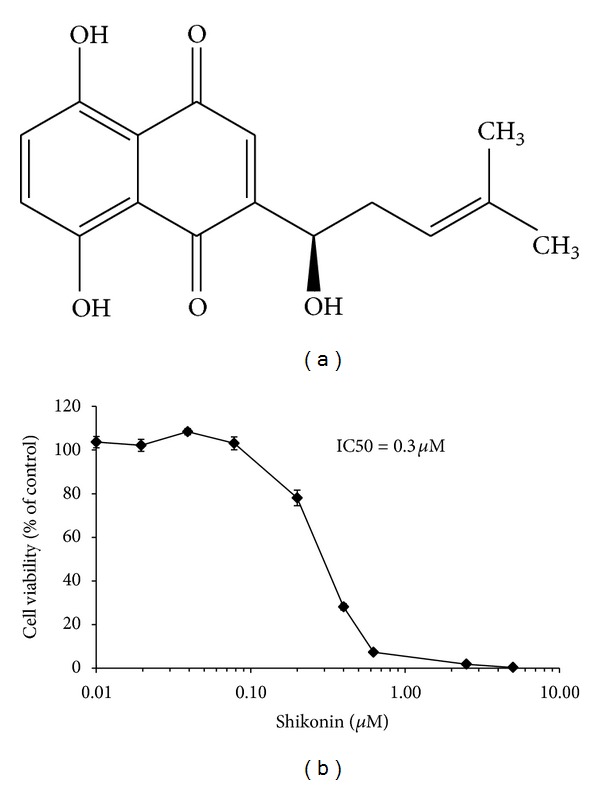
Cytotoxic effect of shikonin against U937 leukemia cells. (a) Chemical structure of shikonin. (b) Dose response curves of U937 cells after treatment with different concentrations of shikonin. Resazurin reduction assays were performed to determine dose response curve. Viability of U937 cells is represented by mean ± SEM of three independent experiments, and it is expressed as percentage survival of control.

The cytotoxic effect of shikonin against U937 leukemia cells was analyzed by resazurin reduction assay. The shikonin dose response curve was calculated after a 24 h treatment of subconfluent U937 cells (Figure 1). Shikonin inhibited U937 proliferation reproducibly by 50% at a concentration of 0.3 *μ*M, and this concentration was subsequently used in experiments for gene expression profiling and stable-isotope dimethyl labeling for quantitative proteomics. 

### 2.2. Omics Data Reveals New Insights into Cellular Mechanisms of Shikonin

**Figure 2:**
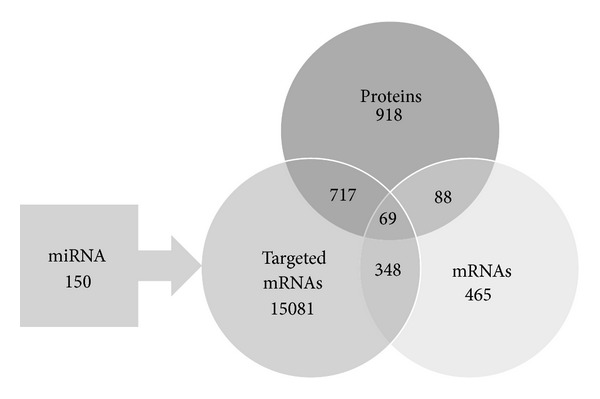
Results and relations of mRNA microarray, miRNA microarray, and stable-isotope dimethyl labeling for quantitative proteomics. Only molecules from the omics datasets that met the expression fold change cutoff of ≥±1.2 are shown in this diagram. The intersections of the Venn diagram indicate the number of corresponding molecules deregulated in different assays independently from the direction of their deregulated expression.

To get deeper insights into the molecular modes of action of shikonin in U937 cells, three “-omics” assays were performed (mRNA microarray, miRNA microarray, and stable-isotope dimethyl labeling for quantitative proteomics) quantifying transcriptomic and proteomic changes after shikonin treatment. The Venn diagram shows the number of deregulated molecules in each assay and the number of predicted mRNA targets of deregulated miRNAs (Figure 2). The intersections of the diagram indicate the number of corresponding molecules deregulated in different assays independently from the direction of their deregulated expression. The overlap of deregulated genes and proteins comprises 88 molecules (19%). Filtered to cases where the corresponding molecules are expressed in the same biological direction, for example up-regulated mRNA levels causing increased protein expression, the total overlap is limited to 52 molecules (11%). The top deregulated molecules are listed in Table 1. 

**Figure 3:**
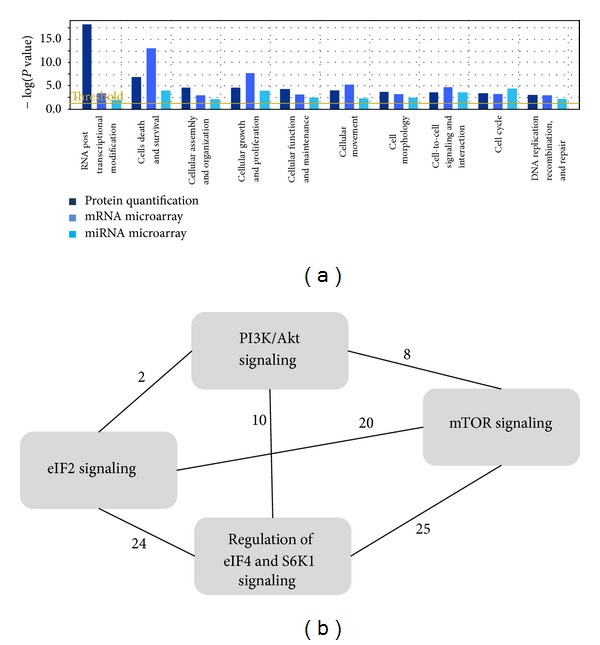
Complementarity of various “-omics” technologies provides a system-level understanding of shikonin's effects in U937 cells. (a) Comparison analysis of molecular and cellular functions affected by shikonin in three “-omics” assays. The bar graph displays only functions disturbed in all three assays. Right-tailed Fisher's exact test was used to calculate a *P* value determining the probability that each biological function assigned to the datasets is due to chance alone. (b) Overlapping signaling pathways deregulated after shikonin treatment. Numbers of identical molecules deregulated in overlapping pathways are shown.

Since each of the three “-omics” assays resulted in a plethora of information about shikonin's cellular mechanisms, we decided to evaluate the datasets in a comparative approach by extracting the most significant results. The data of the single assays were matched using the Ingenuity Pathway Analysis (IPA) comparative analysis tool, revealing cellular functions deregulated by shikonin treatment on the transcriptome as well as on the proteome (Figure 3(a)). The data confirms previous findings indicating that shikonin has strong effects on cell proliferation, cell cycle progression, cellular movement, and DNA integrity of cancer cells. Interestingly, shikonin also affects the posttranscriptional modification of RNA and disturbs cell-to-cell signaling and interaction. The data of the three “-omics” assays were compiled to one data set, and a pathway analysis was performed using IPA. This reanalysis identified a signaling network around the PI3K-Akt-mTOR axis, which was strongly affected by shikonin treatment (Figure 3(b)).

### 2.3. Effect of Shikonin on the PI3K-mTOR Signaling Cascade

**Figure 4:**
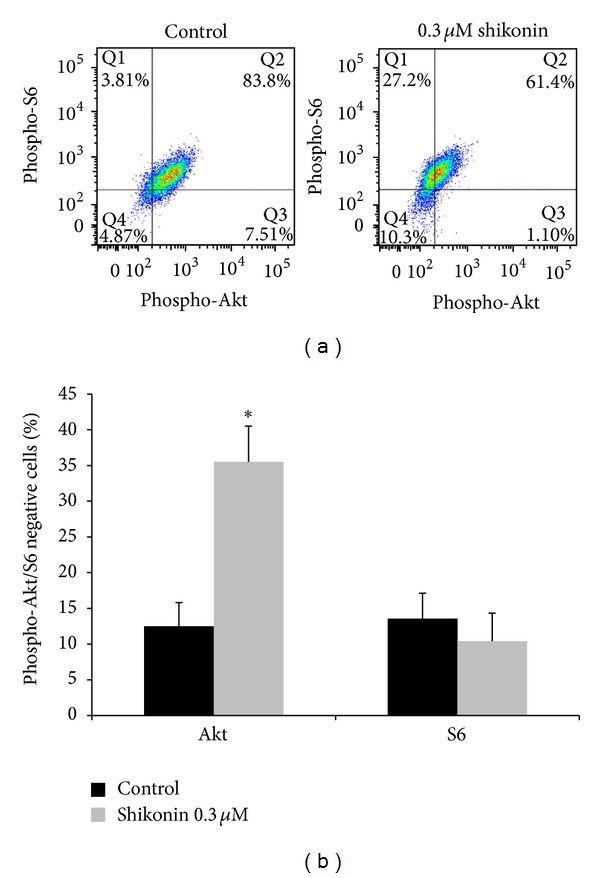
Effect of shikonin on the PI3K-mTOR signaling cascade. (a) U937 cells were treated with 0.3 *μ*M shikonin for 24 h and subsequently stained with phospho-specific antibodies against p-Akt and p-ribosomal protein S6. Shikonin treatment significantly decreased the amount of phosphorylated Akt (left shift), but no effect on the phosphorylation status of ribosomal protein S6 was detected. (b) Statistical quantification of p-Akt or p-ribosomal protein S6 negative cells after shikonin treatment. Data points represent mean ± SD of at least three independent experiments. (*Significantly different according to Student's *t*-test, *P* < 0.05).

We analyzed the PI3K-Akt-mTOR signaling pathway by a close examination of the upstream marker p-Akt and the downstream marker p-ribosomal protein S6 (pRiboS6) by flow cytometry. Samples with and without shikonin treatment were stained with directly conjugated antibodies against p-Akt and pRiboS6. Shikonin significantly inhibits the phosphorylation of Akt, while the phosphorylation of RiboS6 remained almost unchanged (Figure 4). This result indicates an effect of shikonin upstream of the PI3K-mTOR signaling cascade.

### 2.4. Virtual Screening

To identify possible targets of shikonin in the PI3K-mTOR pathway, we conducted a virtual screening approach. Binding energies of shikonin and key proteins of the signaling cascade—with available crystal structures—were calculated using the AutoDock Vina tool [23]. Proteins involved in the PI3K-mTOR pathway were selected according to a recent publication by Laplante and Sabatini [17].  Table 2 summarizes the ranking of the predicted target proteins of shikonin. Within the top potential binding partners, we found the receptor tyrosine kinases EGFR and IGF1R and the Serine/threonine-protein kinase Sgk1 (SGK1). Since the results of the Akt/RiboS6 phosphorylation by FACS showed no effect of shikonin on the phosphorylation status of ribosomal S6 protein, a target of SGK1, we did not further investigate the kinase as target of shikonin. EGFR and IGF1R are both important transmembrane receptors that trigger the PI3K-mTOR signaling cascade after binding to their respective growth factors like EGF, TGF*α*, and IGF. Previous studies showed that shikonin inhibits EGFR phosphorylation and modulates the EGFR signaling cascade [24], and we concentrated on the IGF1R for our further analysis of shikonin action, due to its known evolvement in drug evasion.

### 2.5. Shikonin Inhibits IGF1R kinase Activity

**Figure 5:**
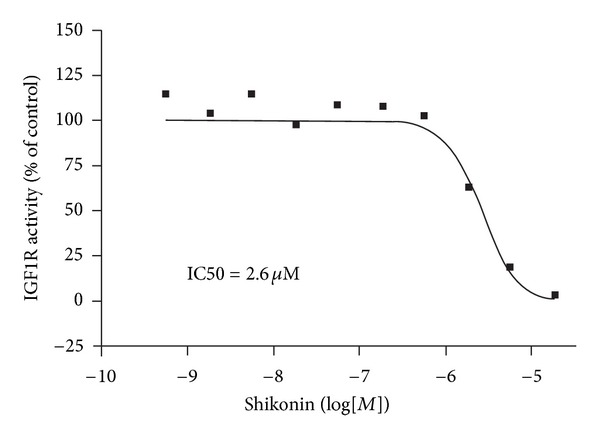
Dose response curve of shikonin in an IGF1-R kinase assay. A radiometric protein kinase assay (33PanQinase Activity Assay) was used for measuring the kinase activity of IGF1R. IC50 values were calculated by nonlinear regression using Prism 5.04 (Graphpad, CA, USA).

Since IGF1R showed a very high binding activity towards shikonin in the virtual screening experiment, we tested the inhibitory effect of shikonin on this kinase using a radiometric protein kinase activity assay. The dose response curve of shikonin on the IGF1R activity clearly indicates a dose-dependent inhibition of the kinase after shikonin application (Figure 5). An IC50 concentration of 2.6 *μ*M was calculated for shikonin on the purified IGF1R kinase activity by nonlinear regression using Prism 5.04 (Graphpad, CA, USA). This IC50 of shikonin on the IGF1R kinase is very similar to the IC50 of about 3 *μ*M observed for U937 cells suggesting that shikonin—besides inducing mitochondrial dysfunction—also exerts additional anticancer activities specifically by inhibiting IGF1R. 

## 3. Discussion

The combination of “-omics” data is a powerful tool to investigate the cellular effects and mechanisms of small molecules [25]. In the present study, we used this experimental approach to show that the natural naphthoquinone shikonin strongly deregulates the IGF1R-Akt-mTOR signaling cascade in U937 myeloid leukemia cells. Three different “-omics” assay indicated that the signaling pathway is disturbed at transcriptomic and proteomic level. Functional validation studies showed that shikonin indeed inhibits two central signaling nodes of the pathway: the kinase activity of IGF1R (a central receptor of the signaling cascade) was inhibited, and in addition the phosphorylation of Akt was significantly decreased upon shikonin application.

The PI3K-Akt-mTOR cascade is the predominant signal transduction pathway after IGF1R activation [15]. Our results are consistent with previous findings indicating that an inhibition of IGF1R causes a decreased phosphorylation and activation of Akt [26]. The reduced Akt phosphorylation in turn causes a decreased activation of the mTOR complexes (mTORC1/2) [12]. mTORC1 regulates cellular protein synthesis by phosphorylation of S6K1 and 4E-BP1, and mTORC2 controls cellular growth and survival through the phosphorylation of many AGC kinases including Akt, SGK1, and PKC-*α* [17]. It was shown that inhibition of critical signaling nodes of this pathway induces cell cycle arrest and apoptosis in leukemia cells [27]. These findings corroborate our results indicating that the cytotoxic effect of shikonin against leukemia cells is reinforced by a direct inhibition of IGF1R and a deregulation of the IGF1R-Akt-mTOR signaling cascade.

The signaling network around the mTOR kinase has been shown to be frequently deregulated in a wide range of hematological malignancies, especially in different types of leukemia [14]. For example, in acute myelogenous leukemia (AML), the mTORC1-signaling pathway is constitutively activated in almost 100% of all patients [28, 29]. Blocking this pathway could be an effective new treatment strategy for leukemia and other hematological malignancies. At the moment, rapamycin and its derivatives (rapalogs), for example, RAD001 (everolimus), CCI-779 (temsirolimus), and AP23573 (deforolimus), are used to partially inhibit the signaling pathway by directly binding the mTORC1 complex [18, 19]. However, results of clinical trials were mostly disappointing and showed only modest anticancer effects of these drugs [14]. A combination of rapalogs with other targeted molecules was more successful, and clinical trials have shown that rapalogs synergize with different conventional chemotherapeutics to overcome resistance [14, 30, 31]. Recent findings showed that a positive feedback loop causes the rapamycin-resistance phenotype: mTOR inhibition by rapalogs leads to an upregulation of IGF1R signaling, which in turn activates the PI3K-Akt-mTOR cascade again [20–22]. Thus, combining an mTOR inhibitor and an IGF-1R inhibitor may be an appropriate strategy to enhance mTOR-targeted anticancer therapy [22]. Since shikonin strongly deregulates the mTOR signaling pathway and in addition directly inhibits the kinase activity of IGF1R, it is a promising candidate for a cotreatment with rapalogs. 

Our results are consistent with recent findings indicating that shikonin also modulates cell proliferation by inhibiting EGFR signaling [24]. EGFR is a further RTK that triggers the PI3K-Akt-mTOR cascade. Results showed that simultaneous targeting of EGFR and mTOR inhibits the growth of cancer cells [32]. An additional inhibition of EGFR signaling makes shikonin even more valuable for targeting the PI3K/mTOR cascade since a second important starting point of the pathway is eliminated. 

Besides the deregulation of the PI3K-Akt-mTOR signaling cascade, we recently showed that shikonin directly targets the mitochondria of cancer cells and thereby triggers apoptosis [7]. Mitochondria targeting drugs were suggested to synergize with the common and clinically established antileukemic drug arsenite trioxide [33]. This makes shikonin even more interesting for the treatment of leukemia. On the one hand, it inhibits the PI3K-mTOR signaling cascade, which is frequently deregulated in leukemia, and in addition it is directly targeting mitochondria of cancer cells, which makes it to a promising candidate for a combined treatment with established antileukemic drugs.

Ultimately, the direct targeting of mitochondria and the simultaneous deregulation of the IGF1R-Akt-mTOR signaling cascade make shikonin a promising compound for the treatment of hematological malignancies.

## 4. Materials and Methods

### 4.1. Chemicals

Shikonin was purchased from Enzo Life Sciences (Lausen, Switzerland) and a 50 mM stock solution was prepared by dissolving it in DMSO. Triethylammonium bicarbonate (TEABC), sodium cyanoborohydride, ammonium hydroxide, formaldehyde (37% solution in H_2_O), formaldehyde-13C, d2 (20% solution in D_2_O), tris (2-carboxyethyl) phosphine hydrochloride (TCEP), ammonium persulfate (APS), methyl methanethiosulfonate (MMTS), Triton X-100 (TX-100), 4-(2-hydroxyethyl) piperazine-1-ethanesulfonic acid (HEPES), sodium bicarbonate, N,N,N′,N′-Tetramethylenediamine (TEMED), 40% acrylamide/bis-acrylamide (37.5 : 1) solution, trifluoroacetic acid (TFA), formic acid (FA), bovine serum albumin (BSA), and bovine beta casein were purchased from Sigma-Aldrich (St. Louis, MO). Ethanol, methanol, acetonitrile (ACN), and sodium dodecyl sulfate (SDS) were purchased from J. T. Baker (Phillipsburg, NJ). Trypsin (modified, sequencing grade) was from Promega (Madison, WI). Deionized water (18.1 MΩ·cm resistivity) from Milli-Q system (Millipore, Bedford, MA) was used throughout this work.

### 4.2. Cell Cultures

U937 cells were obtained from the German Cancer Research Center (DKFZ, Heidelberg, Germany). The original source of the cell line is the American Type Culture Collection (ATCC, USA). U937 cells were maintained in complete RPMI 1640 medium with 2 mM L-glutamine (Invitrogen, Germany) supplemented with 10% FBS (Invitrogen, Germany) and 1% penicillin (100 U/mL)-streptomycin (100 *μ*g/mL) (Invitrogen, Germany). Cells were maintained in a humidified environment at 37°C with 5% CO_2_ and subcultured twice per week. All experiments were performed on cells in the logarithmic growth phase.

### 4.3. Resazurin Reduction Assay

Resazurin reduction assay [34] was performed to assess cytotoxicity of shikonin toward U937 cells. The assay is based on reduction of the indicator dye, resazurin, to the highly fluorescent resorufin by viable cells. Nonviable cells rapidly lose the metabolic capacity to reduce resazurin and thus produce no fluorescent signal. Briefly, aliquots of 2 × 10^4^ U937 cells per well were seeded in 96-well plates in a total volume of 100 *μ*L. Shikonin was immediately added in varying concentrations in an additional 100 *μ*L of culture medium to obtain a total volume of 200 *μ*L/well. After 24 h, 20 *μ*L resazurin (Sigma-Aldrich, Germany) 0.01% w/v in ddH_2_O was added to each well and the plates were incubated at 37°C for 4 h. Fluorescence was measured on an Infinite M2000 Pro plate reader (Tecan, Germany) using an excitation wavelength of 544 nm and an emission wavelength of 590 nm. Each assay was done at least two times, with six replicates each. The viability was evaluated based on a comparison with untreated cells. IC_50_ value represents the shikonin concentrations required to inhibit 50% of cell proliferation and was calculated from a calibration curve by linear regression using Microsoft Excel.

### 4.4. mRNA Microarray

Total RNA from U937 cells after 24 h of treatment with shikonin at IC_50_ concentration or DMSO solvent control was isolated using RNeasy Kit from Qiagen (Hilden, Germany) according to the manufacture's instruction. The quality of total RNA was checked by gel analysis using the total RNA Nano chip assay on an Agilent 2100 Bioanalyzer (Agilent Technologies GmbH, Berlin, Germany). Only samples with RNA index values greater than 9.3 were selected for expression profiling. Microarray experiments were performed in duplicates for treated and untreated samples. Biotin-labeled cRNA samples for hybridization on Illumina Human Sentrix-HT12 Bead Chip arrays (Illumina, Inc.) were prepared according to Illumina's recommended sample labeling procedure based on the modified Eberwine protocol [35]. Biotin-16-UTP was purchased from Roche Applied Science (Penzberg, Germany). The cRNA was column purified with TotalPrep RNA Amplification Kit and eluted in 60 *μ*L of water. Quality of cRNA was controlled using the RNA Nano Chip Assay on an Agilent 2100 Bioanalyzer and spectrophotometrically quantified (NanoDrop). Hybridization was also performed according the manufacturer's recommendations. Microarray scanning was done using a Beadstation array scanner, setting adjusted to a scaling factor of 1 and PMT settings at 430. Data was extracted for each bead individually, and outliers were removed when the MAD (median absolute deviation) was greater than 2.5. Data analysis was performed by using the quantile normalization algorithm without background subtraction, and differentially regulated genes were defined by calculating the standard deviation differences of a given probe in a one-by-one comparison of samples or groups. The expression data obtained was filtered with Chipster data analysis platform. These steps include filtering of genes by two times standard deviation of deregulated genes and a subsequent assessment of significance using empirical Bayes *t*-test (*P* < 0.05).

### 4.5. Real-Time Reverse Transcription PCR

The same RNA samples used in the microarray experiments were also used for RT-PCR experiments. Total RNA samples were converted to cDNA by reverse transcriptase (Invitrogen) with random hexamer primers. Quantification of cDNA was performed by real-time PCR using a Taq-polymerase master mix (Roche) containing the fluorescent dye SYBR Green (Biozol) and the CFX384 Real-Time PCR Detection System (Bio-Rad). The efficiency of all primer pairs used for real-time PCR expression was better than 90%. PCR was performed with an initial denaturation at 95°C for 5 min followed by 50 cycles consisting of strand separation at 95°C for 30 s and annealing and extension at 60°C for 40 s. After PCR product amplification, melting curves were computed. Expression levels were normalized to the transcription level of G6PD. All samples were run in triplicates.

### 4.6. miRNA Microarray

miRNA from U937 cells after 24 h of treatment with shikonin at IC_50_ concentration was isolated using miRNeasy Kit from Qiagen (Hilden, Germany) according to the manufacture's instruction. The quality of miRNA was checked by gel analysis using the Small RNA Nano chip assay on an Agilent 2100 Bioanalyzer (Agilent Technologies GmbH, Germany). The miRNA microarray was performed at the Institute for Molecular Biology (IMB). Microarray experiments were performed in duplicates for treated and untreated samples. Human miRNA microarray chips (8 × 60 K, Agilent Technologies) were used. Probe labeling and hybridization were carried out following the miRNA microarray system with miRNA complete labeling and hyb kit protocol (Agilent Technologies). Briefly, extracted RNA was treated with phosphatase. Dephosphorylated RNA was fluorescently labeled by ligation of Cyanine 3-pCp molecules to the 3′ end of RNA molecules using Agilent's miRNA Complete Labeling and Hyb Kit. The labeled RNA was desalted and hybridized for 20 hours at 55°C and 20 rpm. Microarray slides were washed and scanned with Agilent Microarray Scanning system. Images were analyzed and data were extracted, background subtracted, and normalized using the standard procedures of Agilent Feature Extraction Software. The expression data obtained was filtered with Chipster data analysis platform. These steps include filtering of miRNAs by two times standard deviation of deregulated genes and a subsequent assessment of significance using empirical Bayes *t*-test (*P* < 0.05).

### 4.7. Proteomics Analysis Using Dimethyl Labeling

To examine quantitative changes in the proteome of U937 leukemia cells after shikonin treatment, the dimethyl labeling proteomics analysis was performed. Briefly, 5 × 10^6^ U937 cells were seeded in 10 mL RPMI 1640 medium in a 25 cm^2^ culture flask. Subsequently, cells were treated with 0.3 *μ*M shikonin (IC50) or DMSO as solvent control for 24 h. After incubation cells were washed with PBS and protein extraction was performed using the M-PER Mammalian Protein Extraction Reagent (Thermo Scientific, Germany) containing a protease inhibitor cocktail (Roche, Germany), cells were incubated with the extraction reagent for 40 min at 4°C. After incubation, cellular debris was removed by centrifugation at 14000 ×g for 15 min. Supernatants were transferred into new tubes and an acetone precipitation of the proteins was performed. After precipitation, the acetone was removed and the protein pellet in each of the tube was further diluted to 1 *μ*g/*μ*L with 50 mM TEABC and reduced with 5 mM TCEP for 1 h at 37°C, followed by alkylation using 2 mM MMTS for 45 min at room temperature. For the proteolytic digestion, the modified tube-gel digestion protocol was applied and the detergent residue was checked using the method described previously [36]. Two 200 *μ*g proteolytic protein mixtures from DMSO and shikonin treated cells were first dissolved in 200 *μ*L of 100 mM TEABC (pH 8.5) and, respectively, mixed with 20 *μ*L of formaldehyde and formaldehyde-13C, d2 (4%, diluted with H_2_O). After vortexing (5 min) and centrifugation, each of the sample solutions was mixed with 20 *μ*L of 600 mM sodium cyanoborohydride solution. The sample solutions were vortexed (10 min) and centrifuged again and then allowed to react for 30 min at 25°C. To quench the reaction, ammonium hydroxide (7% in water, 10 *μ*L) was added to each sample solution. Finally, 16 *μ*L of formic acid was added to acidify each of the sample solutions and two samples were further combined for strong cation exchange (SCX) fractionation.

For SCX fractionation, the buffer SCX-A (5 mM KH_2_PO_4_ in 25% ACN at pH 3) and SCX-B (5 mM KH_2_PO_4_ and 350 mM KCl in 25% ACN at pH 3) were used as the mobile phase. The peptide mixtures were reconstituted in buffer SCX-A and then loaded into a PolySULFOETHYL A column (200 × 2.1 mm, 5 *μ*m, 300Å, PolyLC, Columbia, MD) for 10 min at the flow rate of 0.2 mL/min. Peptides were fractionated using a 75 min gradient from 0 to 100% of buffer SCX-B. Fractions were collected every three minutes from the retention time of 10 to 55 min using a fraction collector (BioFrac Fraction Collector, BioRad Laboratories, Hercules, CA). The peptide mixtures in each of the fraction were further analyzed by the LC-MS/MS.

LC-MS/MS analysis was performed with a nanoUHPLC system (nanoACQUITY UPLC, Waters, Millford, MA) coupled online to the nanoelectrospray source of a hybrid quadrupole time-of-flight mass spectrometer (Q-TOF-MS) (SYNAPT HDMS G2, Waters, Manchester, UK). For LC-MS/MS analysis, water with 0.1% FA and ACN with 0.1% FA were used as the mobile phase. The sample was injected into a trap column (Symmetry C18, 5 *μ*m, 180 *μ*m × 20 mm, Waters, Milford, MA) and separated online with a reverse phase column (BEH C18, 1.7 *μ*m, 75 *μ*m × 250 mm, Waters, Milford, MA) at the flow rate of 300 nL/min using either a 95 min gradient with 5–90% ACN/water ratio. The mass spectrometry instruments were all operated in the positive ion mode, and data-dependent acquisition methods were applied for all experiments. The acquisition settings were set to one full MS scan (350–1600 m/z) with a scan time of 1 second and switched to six product ion scans (50–1900 m/z) with 0.4 second scan time when a precursor ion charge was 2+, 3+, or 4+, and the intensity was higher than 200 counts. The data files generated from LC-MS/MS were processed by UniQua and further analyzed by the MASCOT and Trans Proteomics Pipeline (TPP) 1 version 4.4 rev. 1, and the details and criteria of data processing were described previously [37].

### 4.8. Ingenuity Pathway Analysis

“-omics” datasets were analyzed through the use of ingenuity pathway analysis (IPA, Ingenuity Systems, CA, USA; http://www.ingenuity.com/). This software integrates the experimental results to known biological relationships, mechanisms, and functions using the regularly updated Ingenuity Knowledge Base, a giant database of biological findings and relations gathered from the literature. Only molecules from the “-omics” datasets that met the expression fold change cutoff of ≥±1.2 were used for analysis with IPA. Briefly, filtered molecules were fed into Ingenuity Pathway Analysis software, and a separate core analysis was performed for each of the three “-omics” assays to assign the deregulated mRNA, miRNA, and proteins to cellular networks, functions, and pathways. Furthermore, the Ingenuity microRNA Target filter tool was used to associate deregulated miRNAs from the miRNA microarray with experimentally observed and predicted mRNA targets. The results of the core analysis were further studied in a comparison analysis to identify cellular functions affected in all three assays. Finally, the results of the “-omics” assays were merged in one dataset that was screened for overlapping cellular signaling pathways using the core analysis tool. For molecules that showed a nonconsistent deregulation of mRNA and protein expression (36 cases), the stronger deregulated expression values were considered for analysis.

### 4.9. FlowCellect PI3K-mTOR Signaling Cascade Assay

The FlowCellect PI3K-mTOR signaling cascade assay kit (Merck Millipore, Germany) was used to analyze the effect of shikonin on the PI3K-mTOR signaling pathway. The assay is based on two directly conjugated phospho-specific signaling antibodies against phosphorylated Akt and phosphorylated ribosomal S6 protein, which are both important signaling nodes of the mTOR cascade. The phosphorylation of Akt is indicative of the upstream PI3K signaling, marking the cells initiation into proliferation or cell survival. Phosphorylated-ribosomal S6 protein is indicative of downstream mTOR and p70S6K signaling leading to protein translation. The assay was performed according to the manufacture's protocol. Briefly, 1.5 × 10^6^ U937 cells were treated with 0.3 *μ*M shikonin or DMSO solvent control and incubated for 24 h. Subsequently, cells were fixed on ice for 20 min using the supplied fixation buffer. After fixation, cells were washed twice and treated with permeabilization buffer on ice for 20 min. After two further washing steps, cells were resuspended in a final volume of 90 *μ*L assay buffer and incubated with 5 *μ*L 20x anti-phospho-ribosomal protein S6 (Ser235) PerCP conjugated monoclonal antibody and 5 *μ*L 20x anti-phospho-Akt1/PKB*α* (Ser473) Alexa Fluor 488 conjugated monoclonal antibody in the dark on ice for one hour. After antibody incubation, cells were centrifuged and resuspended in 500 *μ*L assay buffer. Subsequently, cells were measured in a LSR-Fortessa FACS analyzer (Becton-Dickinson, Germany) and shikonin fluorescence was compensated for the respective channels. For each sample, 1 × 10^4^ cells were counted. The PerCP signal was measured with 488 nm excitation and detected using a 670/30 nm bandpass filter. The Alexa Fluor 488 signal was analyzed with 488 nm excitation and detected using a 530/30 nm bandpass filter. All parameters were plotted on a logarithmic scale. Cytographs were analyzed using FlowJo software (Celeza, Switzerland). All experiments were performed at least in triplicate. Student's *t*-test was used for statistical analysis.

### 4.10. Virtual Screening

Virtual screening represents an approach to identify possible binding interactions between ligands and proteins without any previous knowledge of interactions or binding sites. Since shikonin deregulates the PI3K-mTOR signaling cascade, a virtual screening approach was used to identify proteins of the mTOR pathway which are most likely targeted by shinonin. Virtual screening was performed using AutoDock Vina with the graphical user interface AutoDock Tools [23]. X-ray structures of proteins involved in signaling cascade were downloaded from the “Protein Data Bank” (http://www.pdb.org/). The three-dimensional structure of shikonin was downloaded from the PubChem compound library (http://pubchem.ncbi.nlm.nih.gov/). 

### 4.11. IGF1R Kinase Inhibition Assays

The kinase inhibition assay was performed by ProQinase GmbH (Freiburg, Germany). Shikonin was provided as 1.85 × 10^−3^ M stock solution in DMSO. In the process, shikonin was serially diluted in semi-log steps with 100% DMSO in a 96-well microtiter plate. Directly before use, shikonin was further diluted 1 : 10 with water. Shikonin was tested at 10 final assay concentrations in the range from 1.85 × 10^−5 ^M to 5.55 × 10^−10 ^M. The final DMSO concentration in the reaction cocktails was 1% in all cases. A radiometric protein kinase assay (^33^PanQinase Activity Assay) was used for measuring the kinase activity of IGF1R. All kinase assays were performed in 96-well FlashPlates from Perkin Elmer (Boston, MA, USA) in a 50 *μ*L reaction volume. The reaction cocktail was pipetted in 4 steps in the following order: 10 *μ*L of nonradioactive ATP solution (in H_2_O), 25 *μ*L of assay buffer/[*γ*-^33^P]-ATP mixture, 5 *μ*L shikonin in 10% DMSO, and 10 *μ*L of IGF1R/Poly(Glu, Tyr)4 : 1 mixture. The reaction cocktails were incubated at 30°C for 60 minutes. The reaction was stopped with 50 *μ*L of 2% (v/v) H_3_PO_4_, and plates were aspirated and washed two times with 200 *μ*L 0.9% (w/v) NaCl. Incorporation of ^33^P_i_ (counting of “cpm”) was determined with a Wallac MicroBeta scintillation counter (Perkin Elmer). All assays were performed with a Beckman Coulter Biomek 2000/SL robotic system. Kinase activity was evaluated based on a comparison with experiments containing the complete reaction cocktail but no shikonin. A complete reaction cocktail without kinase served as control for unspecific binding of radioactivity to the plate in the absence of protein kinase but in the presence of shikonin. IC50 values were calculated by nonlinear regression using Prism 5.04 (Graphpad, CA, USA).

## Figures and Tables

**Table 1:** Top up- and downregulated molecules in U937 cells after treatment with shikonin. Quantitative changes in the proteome were studied by stable-isotope dimethyl labeling. Microarray experiments were used to analyze changes in the mRNA and miRNA expressione.

	Proteins		mRNAs			miRNAS	
	Protein names	FC	Genes	Encoded proteins	FC	ID	FC
Top upregulated molecules	Heme oxygenase 1	+14.3	MLLT11	Protein AF1q	+6.2	miR-19b-2-5p	+5.2
	WD repeat-containing protein 3	+12.1	S100A8	Protein S100-A8	+4.7	miR-20b-3p	+3.7
	Histone H4	+11.1	LY96	Lymphocyte antigen 96	+4.5	miR-155-3p	+3.4
	Cell differentiation protein RCD1 homolog	+10.0	S100A9	Protein S100-A9	+4.2	miR-181a-2-3p	+3.2
	Sorting nexin-17	+9.1	CCL2	C-C motif chemokine 2	+4.2	miR-33b-5p	+3.1
	Mannose-6-phosphate isomerase	+7.7	VIM	Vimentin	+4.2	miR-30a-3p	+2.9
	Histone H2B type 2-E	+7.1	HMOX1	Heme oxygenase 1	+4.1	miR-3907	+2.8
	Schlafen family member 11	+6.7	ANXA1	Annexin A1	+4.1	miR-223-5p	+2.7
	LIM domain and actin-binding protein 1	+6.7	MAFB	Transcription factor MafB	+3.5	miR-193b-3p	+2.7
	Neuronal-specific septin-3	+6.7	JUN	Transcription factor AP-1	+3.2	miR-92a-3p	+2.7

Top downregulated molecules	Hexokinase-1	−20.5	ALDOA	Fructose-bisphosphate aldolase A	−17.6	miR-4299	−30.6
	Vacuolar protein sorting-associated protein 11 homolog	−20.0	ACTG1	Actin, cytoplasmic 2	−11.9	miR-1915-3p	−13.8
	60S ribosomal protein L24	−7.5	CCT7	T-complex protein 1 subunit eta	−7.8	miR-2861	−9.8
	5′-Nucleotidase domain-containing protein 2	−5.4	ARPC1B	Actin-related protein 2/3 complex subunit 1B	−7.8	miR-1207-5p	−6.3
	Heterogeneous nuclear ribonucleoprotein H2	−4.5	PSMC4	26S protease regulatory subunit 6B	−7.7	miR-1290	−6.2
	ATP-dependent RNA helicase DDX3Y	−4.3	ACTB	Actin, cytoplasmic 1	−6.8	miR-638	−6.1
	Methylosome protein 50	−4.0	HSPD1	60 kDa heat shock protein, mitochondrial	−6.6	miR-1246	−6.0
	Golgi phosphoprotein 3	−3.5	SLC39A3	Zinc transporter ZIP3	−6.2	miR-1185-5p	−5.2
	Leucine-rich repeat-containing protein 58	−3.5	ERP29	Endoplasmic reticulum resident protein 29	−6.0	miR-630	−5.1
	Rho GTPase-activating protein 4	−3.1	NCF1	Neutrophil cytosol factor 1	−5.9	miR-513a-5p	−4.7

**Table 2:** Calculation of binding energies of shikonin and key proteins of the IGF1R-Akt-mTOR signaling pathway. Virtual screening was performed using the AutoDock Vina software.

Symbol	Description	Binding energy [kcal/mol]
EGFR	Epidermal growth factor receptor	−8.8
SGK1	Serine/threonine-protein kinase Sgk1	−8.8
IGF1R	Insulin-like growth factor 1 receptor	−8.6
GSK3B	Glycogen synthase kinase-3 beta	−8.5
4E-BP1	Eukaryotic translation initiation factor 4E-binding protein 1	−8.5
S6K1	Ribosomal protein S6 kinase beta-1	−8.4
PKCA	Protein kinase C alpha type	−8.4
PIK3C3	Phosphatidylinositol 3-kinase catalytic subunit type 3	−8.3
PDK1	3-phosphoinositide-dependent protein kinase 1	−8.3
eIF4E	Eukaryotic translation initiation factor 4E	−8.3
RND3	Rho-related GTP-binding protein RhoE	−8.2
AKT2	RAC-beta serine/threonine-protein kinase	−8.1
RSK1	Ribosomal protein S6 kinase alpha-1	−8.1
Rheb	GTP-binding protein Rheb	−7.9
AMPK2	5′-AMP-activated protein kinase catalytic subunit alpha-2	−7.9
RHOA	Transforming protein RhoA	−7.9
AKT1	RAC-alpha serine/threonine-protein kinase	−7.8
RRAGD	Ras-related GTP-binding protein D	−7.8
mTOR	Serine/threonine-protein kinase mTOR	−7.5
RHOC	Rho-related GTP-binding protein RhoC	−7.3
RHOB	Rho-related GTP-binding protein RhoB	−7.2
ERK1	Extracellular signal-regulated kinase 1	−7.1
RHOD	Rho-related GTP-binding protein RhoD	−6.9
IRS1	Insulin receptor substrate 1	−6.4
IKKB	Inhibitor of nuclear factor kappa-B kinase subunit beta	−5.1

## Abbreviations

ACN: Acetonitrile
AML: Acute myelogenous leukemia
AP23573: Deforolimus
APS: Ammonium persulfate
BSA: Bovine serum albumin
CCI-779: Temsirolimus
EGFR: Epidermal growth factor receptor
4E-BP1: 4E-binding protein
FA: Formic acid
FC: Fold change
HEPES: 4-(2-Hydroxyethyl) piperazine-1-ethanesulfonic acid
IC50: Half maximal inhibitory concentration
IGF1R: Insulin-like growth factor 1 receptor
IPA: Ingenuity Pathway Analysis
LC-MS: liquid chromatography-mass spectrometry
MMTS: Methyl methanethiosulfonate
MS: Mass spectrometry
mTOR: Mammalian target of rapamycin
mTORC1/2: Mammalian target of rapamycin complex 1 and 2
PI3K: Phosphatidylinositol 3-kinase
Q-TOF-MS: Quadrupole time-of-flight mass spectrometer
RAD001: Everolimus
RiboS6: Ribosomal protein S6
ROS: Reactive oxygen species
RTK: Receptor tyrosine kinase
S6K1: S6 kinase 1
SCX: Strong cation exchange
SDS: Sodium dodecyl sulfate
SGK1: Serine/threonine-protein kinase Sgk1
TCM: Traditional Chinese medicine
TEABC: Triethylammonium bicarbonate
TCEP: Tris (2-carboxyethyl) phosphine hydrochloride
TEMED: N,N,N′,N′-Tetramethylenediamine
TFA: Trifluoroacetic acid
TPP: Trans-proteomics pipeline
TSC1/2: Tuberous sclerosis proteins 1 and 2
TX-100: Triton X-100.
